# Supplementary figures and images for: Reduction of Derlin activity suppresses Notch-dependent tumours in the C. elegans germ line
Source: PLoS Genet. 2021 Sep 23;17(9):e1009687. doi: 10.1371/journal.pgen.1009687 (PMC8491880; doi:10.1371/journal.pgen.1009687)

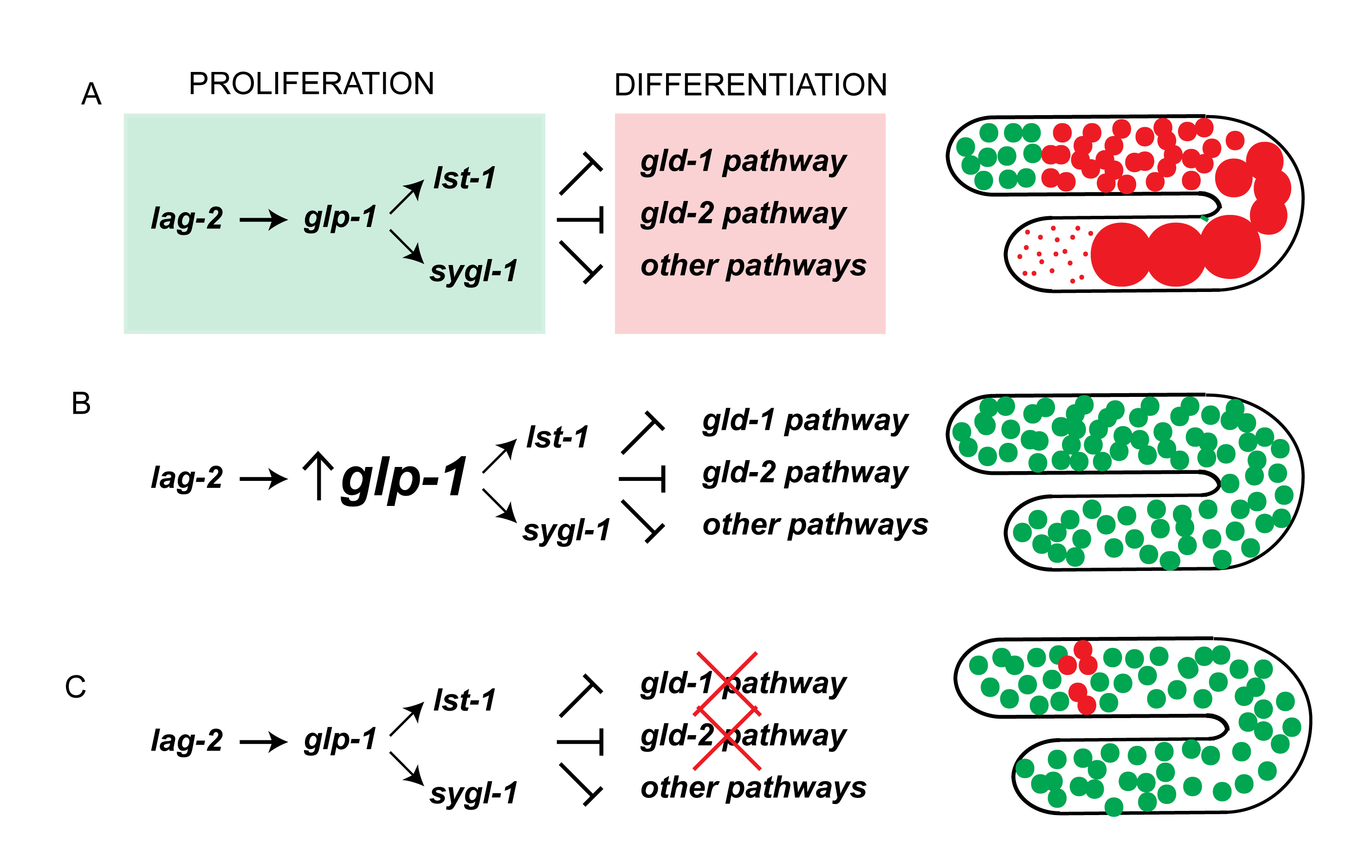

Supplement: S1 Fig — (A) Wild-type gonad. (B) glp-1(gf) gonad (C) gld-2(0) gld-1(0) gonad. Proliferative cells in green, differentiating cells in red within the tube-like gonad. (TIF) [file pgen.1009687.s001.tif]

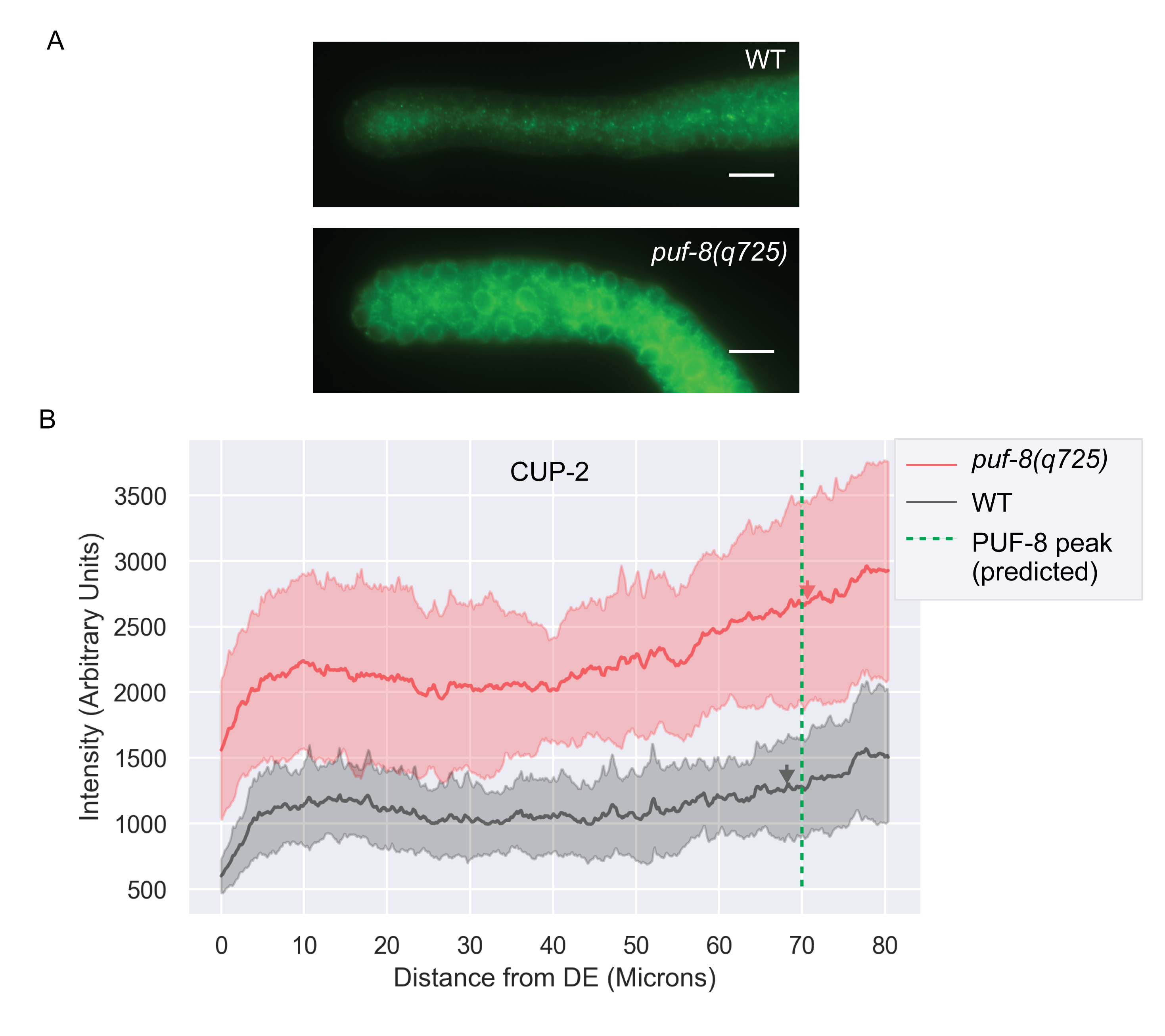

Supplement: S2 Fig — (A). CUP-2 immunostaining (by α-V5) in wild-type (WT) and puf-8(q725) dissected gonads. Both strains contain the cup-2(ug1[V5::2XFLAG::CUP-2]) allele. Scale bar = 10μm. (B) CUP-2 intensities measured by α-V5 immunostaining by drawing a line through the center of the germline from the distal end along the distal-proximal axis. Shaded area represents the standard deviation of average intensity measurements of each genotype. Fifteen germlines were analyzed for CUP-2 intensity measurements of each genotype. Arrowheads point to the average location of the transition zone measured in at least seven gonads of each genotype. Dashed line represents the predicted peak of PUF-8 expression, based on previous work that found that PUF-8’s expression pattern is a bell-shaped curve centered around the transition zone with low expression levels in the distal end [54]. While we find that the overall CUP-2 expression levels are higher in puf-8(q725) gonads compared to wild type gonads, since this increase does not correlate with the known expression pattern of PUF-8 in wild type gonads, CUP-2 levels are unlikely to be directly regulated by PUF-8. (TIF) [file pgen.1009687.s002.tif]

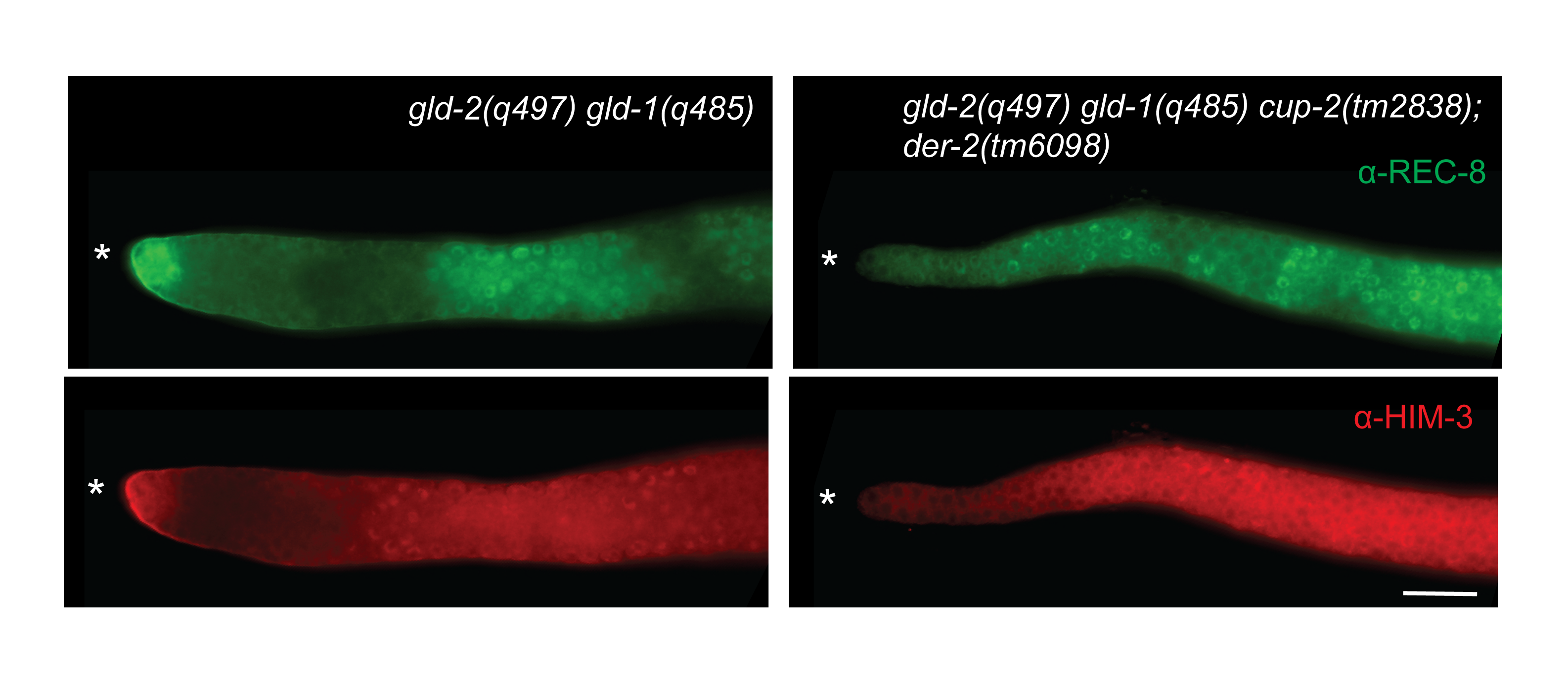

Supplement: S3 Fig — Asterisk, distal tip. Scale bar = 20μm (TIF) [file pgen.1009687.s003.tif]

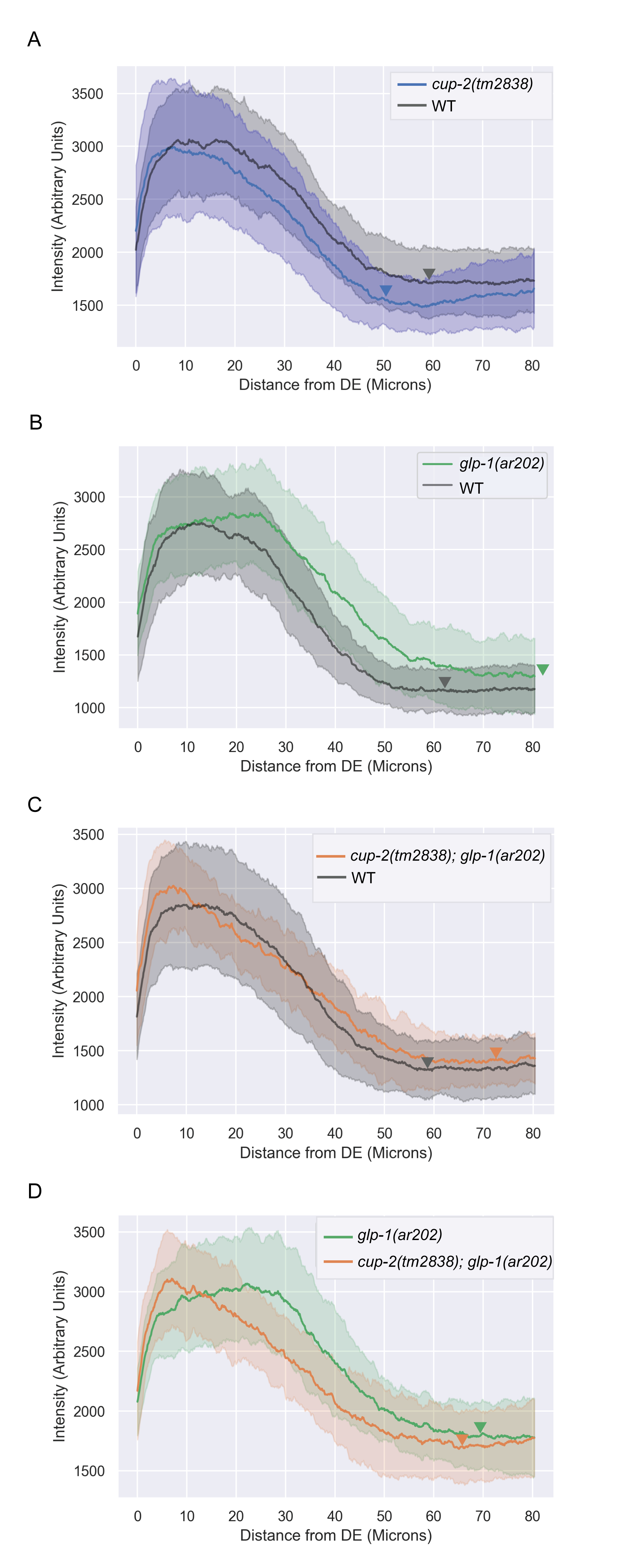

Supplement: S4 Fig — SYGL-1 intensities were measured by α-FLAG immunostaining by drawing a line through the center of the germline from the distal end along the distal-proximal axis of the indicated genotypes. Each subfigure indicates an individual experiment comparing two genotypes that were processed together and imaged on the same slide with the same exposure setting. A-C measurements were used to generate scaled, fitted intensity curves shown in Fig 4B. Shaded area represents the standard deviation of average intensity measurements of each genotype. Fifteen germlines were analyzed for SYGL-1 intensity measurements of each genotype. Arrowheads point to the average location of the transition zone measured in at least seven gonads of each genotype. A. Average SYGL-1 intensity comparison of sygl-1(am307) against cup-2(tm2838) sygl-1(am307) germlines. B. Average SYGL-1 intensity comparison of sygl-1(am307) against sygl-1(am307); glp-1(ar202) germlines. C. Average SYGL-1 intensity comparison of sygl-1(am307) against cup-2(tm2838) sygl-1(am307); glp-1(ar202) germlines. D. Average SYGL-1 intensity comparison of sygl-1(am307); glp-1(ar202) against cup-2(tm2838) sygl-1(am307); glp-1(ar202) germlines (TIF) [file pgen.1009687.s004.tif]

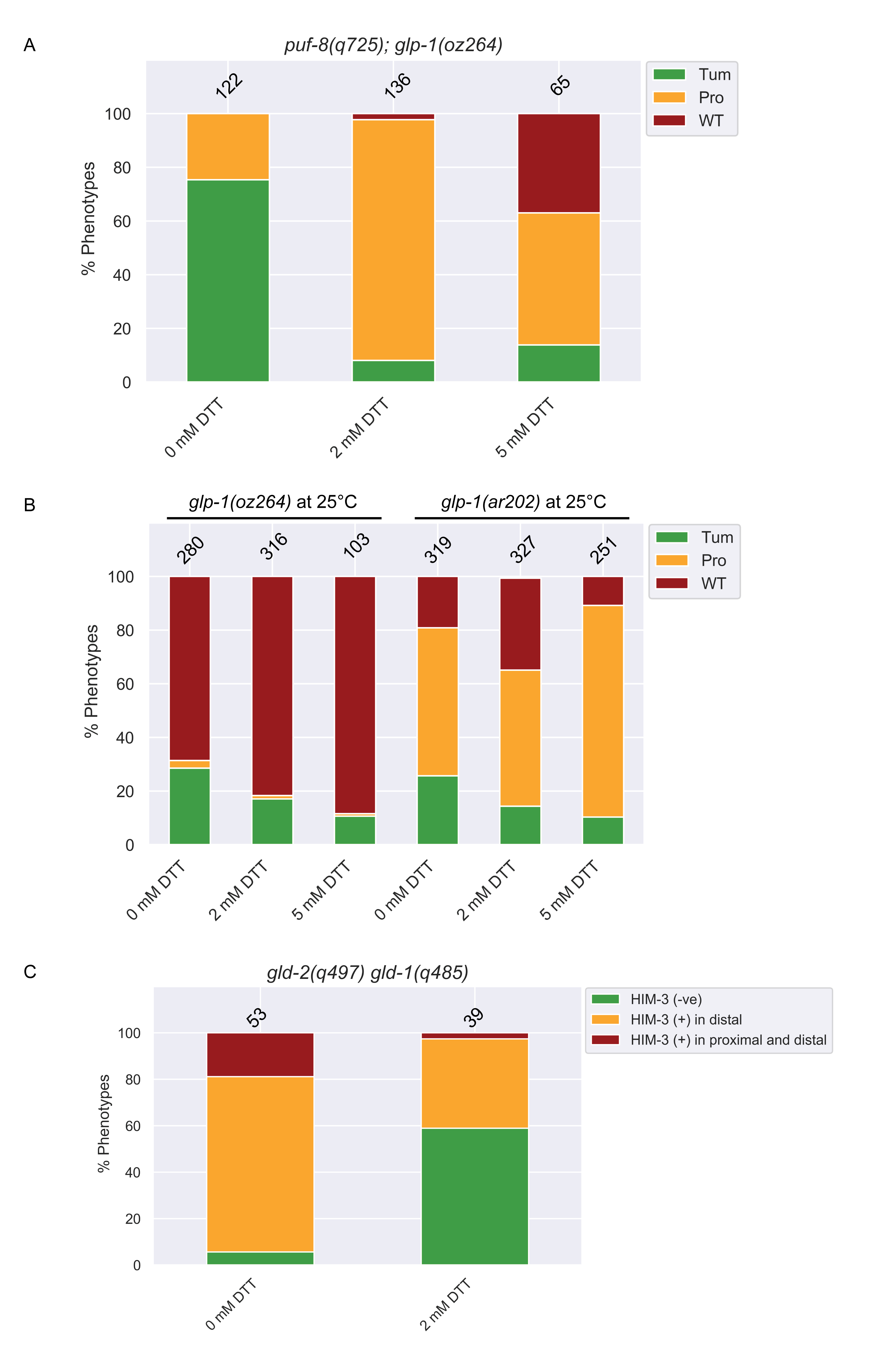

Supplement: S5 Fig — A. Suppression of puf-8(q725); glp-1(oz264) tumours by DTT treatment. Phenotypes were analyzed by dissections followed by α-REC-8/α-HIM-3 staining. B. Quantification of phenotypic analysis of the effect of increasing doses of DTT on glp-1(oz264) and glp-1(ar202) tumours at 25°C. Phenotypes were analyzed by whole mount DAPI. C. Quantification of phenotypic analysis of the effect of increasing doses of DTT on Notch-independent gld-2(q497) gld-1(q485) tumours. Phenotypes were analyzed by dissections followed by α-REC-8/α-HIM-3 staining. (TIF) [file pgen.1009687.s005.tif]

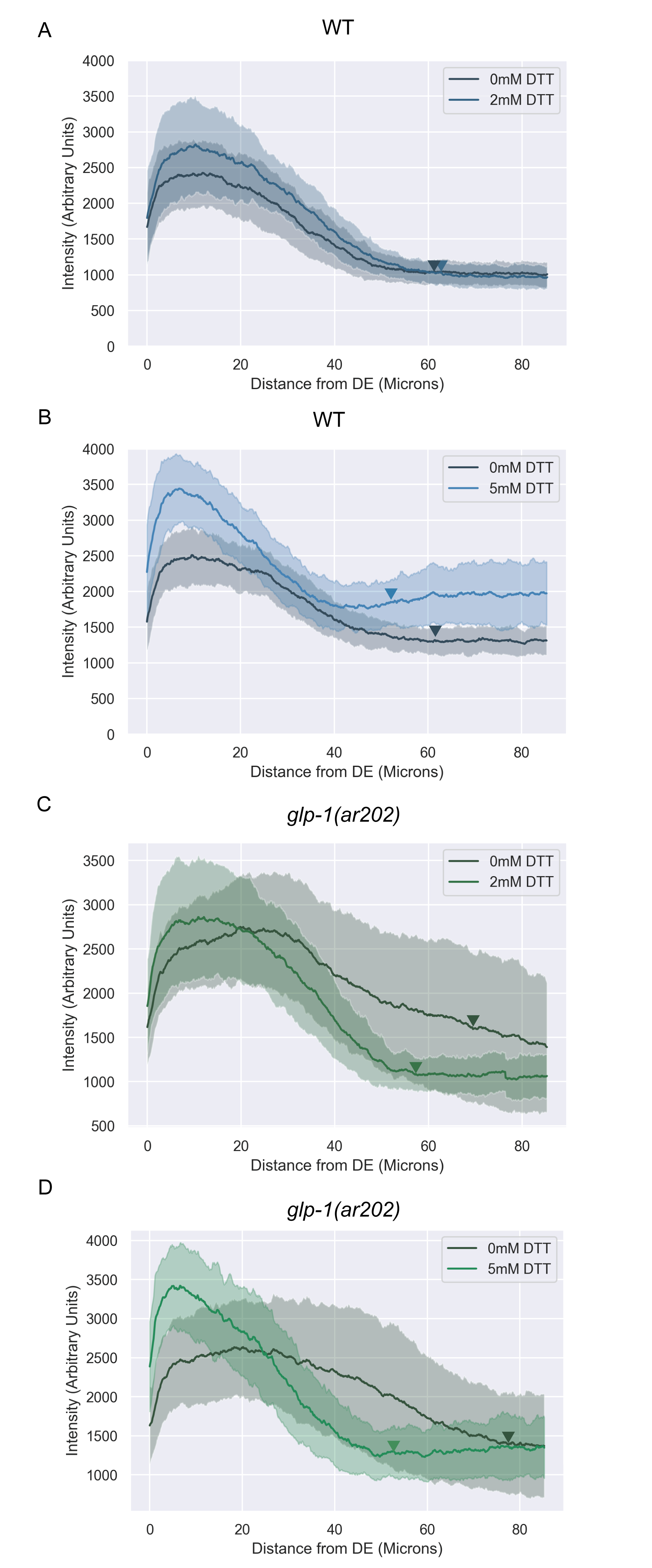

Supplement: S6 Fig — SYGL-1 intensities were measured by α-FLAG immunostaining by drawing a line through the center of the germline from the distal end along the distal-proximal axis of the indicated treatments. Each subfigure indicates an individual experiment comparing two treatments that were processed together and imaged on the same slide with the same exposure setting. A-B measurements were used to generate scaled, fitted intensity curves shown in Fig 8A, C-D measurements were used to generate scaled, fitted intensity curves shown in Fig 8B. Shaded area represents the standard deviation of average intensity measurements of each treatment. Fifteen germlines were analyzed for SYGL-1 intensity measurements of each treatment. Arrowheads point to the average location of the transition zone measured in at least seven gonads of each treatment. A. Average SYGL-1 intensity comparison of 0 DTT treated against 2 DTT treated sygl-1(am307) germlines. B. Average SYGL-1 intensity comparison of 0 DTT treated against 5 DTT treated sygl-1(am307) germlines. C. Average SYGL-1 intensity comparison of 0 DTT treated against 2 DTT treated sygl-1(am307); glp-1(ar202) germlines. D. Average SYGL-1 intensity comparison of 0 DTT treated against 5 DTT treated sygl-1(am307); glp-1(ar202) germlines. (TIF) [file pgen.1009687.s006.tif]

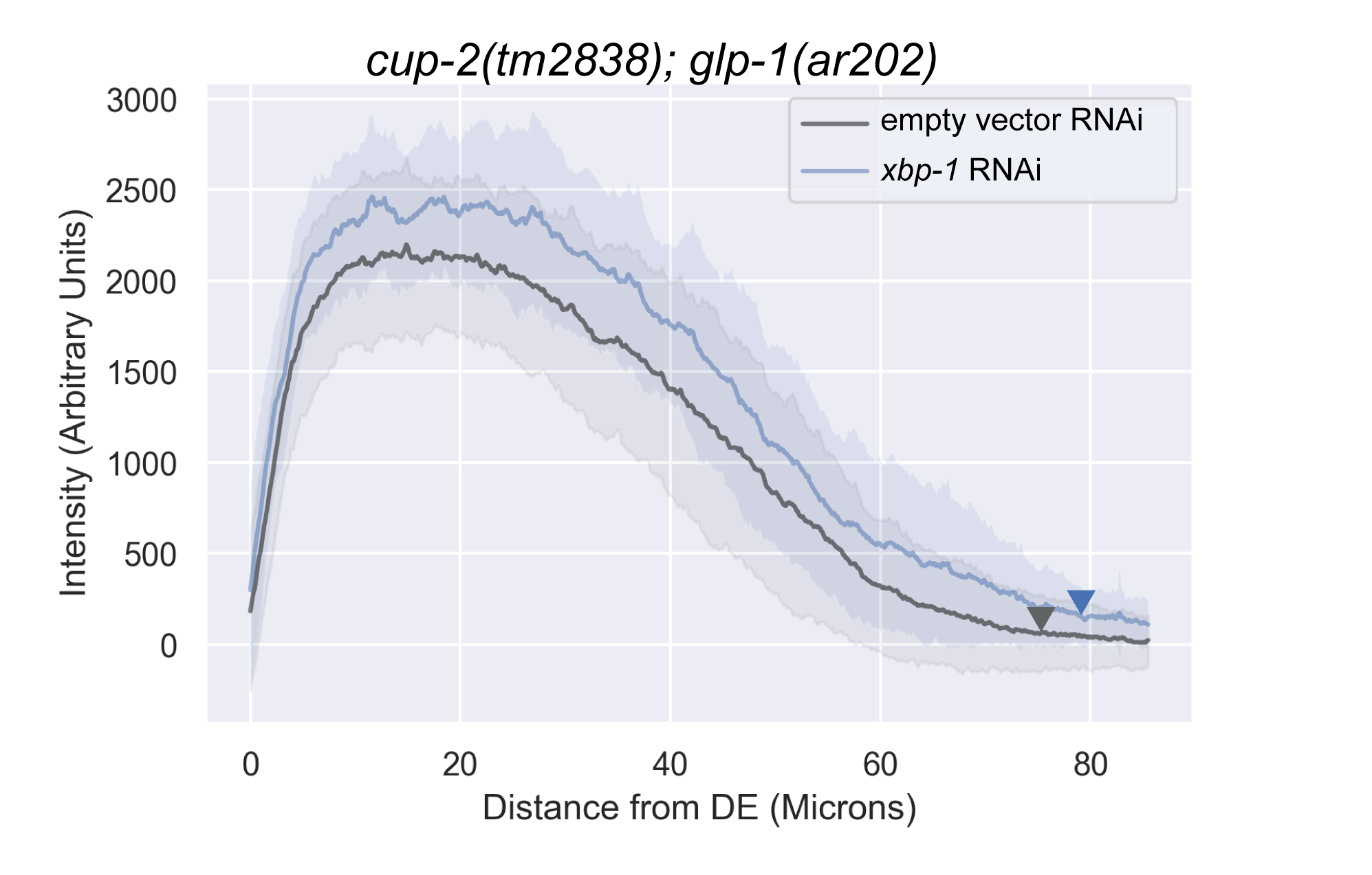

Supplement: S7 Fig — SYGL-1 intensities were measured by α-FLAG immunostaining by drawing a line through the center of the germline from the distal end along the distal-proximal axis of the indicated treatments. Shaded area represents the standard deviation of average intensity measurements of each treatment. At least twelve gonads were analyzed for SYGL-1 intensity measurements of each treatment. (TIF) [file pgen.1009687.s007.tif]
