# Supplementary material for: Reduction of Derlin activity suppresses Notch-dependent tumours in the C. elegans germ line
Source: PLoS Genet. 2021 Sep 23;17(9):e1009687. doi: 10.1371/journal.pgen.1009687 (PMC8491880; doi:10.1371/journal.pgen.1009687)
Supplement: S1 Table — (DOCX) [file pgen.1009687.s008.docx]

**S1 Table** – **Key *glp-1* alleles used in this study**

| Allele | Location | Nature | References |
| --- | --- | --- | --- |
| *oz264* | G528E,  extracellular domain | temperature sensitive gain-of-function | [1,2] |
| *ar202* | G529E,  extracellular domain | temperature sensitive gain-of-function | [3] |
| *ar224* | A729T,  extracellular domain | temperature sensitive gain-of-function | [3] |
| *bn18* | A1034T,  intracellular domain | temperature sensitive loss-of-function | [4] |

**Reference**

1. Kerins JA. PRP-17 and the pre-mRNA splicing pathway are preferentially required for the proliferation versus meiotic development decision and germline sex determination in Caenorhabditis elegans. Wahington University, St. Louis, MO. 2006.

2. Kerins JA, Hanazawa M, Dorsett M, Schedl T. PRP-17 and the pre-mRNA splicing pathway are preferentially required for the proliferation versus meiotic development decision and germline sex determination in Caenorhabditis elegans. Developmental Dynamics. 2010;239: 1555–1572. doi:10.1002/dvdy.22274

3. Pepper ASR, Killian DJ, Hubbard EJA. Genetic analysis of Caenorhabditis elegans glp-1 mutants suggests receptor interaction or competition. Genetics. 2003;163: 115–132.

4. Kodoyianni V, Maine EM, Kimble J. Molecular Basis of Loss-of-Function Mutations in the glp-1 Gene of Caenorhabitis elegans. Molecular Biology of the Cell. 1992;3: 1199–1213. doi:10.1083/jcb.118.3.753
