# Supplementary material for: Reduction of Derlin activity suppresses Notch-dependent tumours in the C. elegans germ line
Source: PLoS Genet. 2021 Sep 23;17(9):e1009687. doi: 10.1371/journal.pgen.1009687 (PMC8491880; doi:10.1371/journal.pgen.1009687)
Supplement: S2 Table — Individual gonads were analyzed at 22.5°C by whole mount DAPI. (DOCX) [file pgen.1009687.s009.docx]

**S2 Table - Phenotypic analysis of the effect of loss of *cup-2* on expression of the Glp phenotype.** Individual gonads were analyzed at 22.5°C by whole mount DAPI.

| Genotype | % Glp | n |
| --- | --- | --- |
| *glp-1(bn18)* | 23.5 % | 294 |
| *cup-2(tm2838); glp-1(bn18)* | 20.35 % | 314 |
