# Supplementary material for: Reduction of Derlin activity suppresses Notch-dependent tumours in the C. elegans germ line
Source: PLoS Genet. 2021 Sep 23;17(9):e1009687. doi: 10.1371/journal.pgen.1009687 (PMC8491880; doi:10.1371/journal.pgen.1009687)
Supplement: S3 Table — puf-8(q725); glp-1(oz264) was analyzed by dissections followed by α-REC-8/α-HIM-3 staining while the glp-1(oz264) and glp-1(ar202) phenotypes was analyzed by whole mount DAPI. (DOCX) [file pgen.1009687.s010.docx]

**S3 Table- Phenotypic analysis of the effect of ER stress induced by DTT treatment on suppression of Notch-dependent tumorous phenotypes.** *puf-8(q725); glp-1(oz264)* was analyzed by dissections followed by α-REC-8/α-HIM-3 staining while the *glp-1(oz264)* and *glp-1(ar202)* phenotypes was analyzed by whole mount DAPI.

| Genotype | Temperature | Treatment | WT | Protumour | Complete Tumour | n |
| --- | --- | --- | --- | --- | --- | --- |
| *puf-8(q725); glp-1(oz264)* | 20°C | 0 mM DTT | 0% | 25% | 75% | 122 |
|  |  | 2 mM DTT | 2% | 90% | 8% | 136 |
|  |  | 5 mM DTT | 37% | 49% | 14% | 65 |
| *glp-1(oz264)* | 25°C | 0 mM DTT | 69% | 3% | 28% | 280 |
|  |  | 2 mM DTT | 82% | 1% | 17% | 316 |
|  |  | 5 mM DTT | 88% | 1% | 11% | 103 |
| *glp-1(ar202)* | 25°C | 0 mM DTT | 19% | 55% | 26% | 319 |
|  |  | 2 mM DTT | 34% | 51% | 15% | 327 |
|  |  | 5 mM DTT | 11% | 79% | 10% | 251 |
