# Supplementary material for: Reduction of Derlin activity suppresses Notch-dependent tumours in the C. elegans germ line
Source: PLoS Genet. 2021 Sep 23;17(9):e1009687. doi: 10.1371/journal.pgen.1009687 (PMC8491880; doi:10.1371/journal.pgen.1009687)
Supplement: S4 Table — Phenotypes were analyzed by dissections followed by α-REC-8/α-HIM-3 staining. (DOCX) [file pgen.1009687.s011.docx]

**S4 Table - Phenotypic analysis of the effect of ER stress induced by DTT treatment on suppression of a *gld-2 gld-1* Notch-independent tumours.** Phenotypes were analyzed by dissections followed by α-REC-8/α-HIM-3 staining.

| Genotype | Treatment | HIM-3(-) | HIM-3(+) in proximal germline | HIM-3(+) in proximal and distal germline | n |
| --- | --- | --- | --- | --- | --- |
| *gld-2(q497) gld-1(q485)* | 0 mM DTT | 6% | 75% | 19% | 53 |
|  | 2 mM DTT | 59% | 38% | 3% | 39 |
