# Supplementary material for: Reduction of Derlin activity suppresses Notch-dependent tumours in the C. elegans germ line
Source: PLoS Genet. 2021 Sep 23;17(9):e1009687. doi: 10.1371/journal.pgen.1009687 (PMC8491880; doi:10.1371/journal.pgen.1009687)
Supplement: S5 Table — (DOCX) [file pgen.1009687.s012.docx]

**S5 Table: List of strains used**

| **Strain** | **Genotype** |
| --- | --- |
| N2 | Wild type |
| FX02838 | *cup-2(tm3838) I* |
| FX06098 | *der-2(tm6098) II* |
| XB671 | *cup-2(tm2838)/hT2g I ; der-2(tm6098)/hT2g III* |
| BS3148 | *glp-1(ar202) III* |
| XB590 | *cup-2(tm2838) I; glp-1(ar202) III* |
| XB655 | *der-2(tm6098) glp-1(ar202) III* |
| XB659 | *cup-2(tm2838)/hT2g I; der-2(tm6098) glp-1(ar202)/hT2g III* |
| BS121 | *glp-1(bn18) III* |
| XB663 | *cup-2(tm2838) I; glp-1(bn18) III* |
| XB739 | *cup-2(tm2838)/hT2g I; unc-32(e189) glp-1(ar224)/hT2g III* |
| XB740 | *unc-32(e189) glp-1(ar224) / hT2g III* |
| XB184 | *glp-1(oz264) III* |
| XB492 | *cup-2(tm2838) I; glp-1(oz264) III* |
| XB445 | *rfp-1(ok572) glp-1(oz264)/hT2g III* |
| XB661 | *cup-2(tm2838)/hT2g I; rfp-1(ok572) glp-1(oz264)/hT2g III* |
| XB745 | *puf-8(q725)/mc6g II; unc-32(189) glp-1(ar224) III* |
| XB744 | *cup-2(tm2838) I; puf-8(q725)/mc6g II; unc-32(e189) glp-1(ar224) III* |
| XB306 | *puf-8(q725)/mC6g II; glp-1(oz264) III* |
| XB506 | *cup-2(tm2838) I; puf-8(q725)/mC6g II; glp-1(oz264) III* |
| XB269 | *puf-8(q725)/mC6g II; unc-32(e189) glp-1(ar202)/hT2 III* |
| XB591 | *cup-2(tm2838) I; puf-8(q725)/mC6g II; glp-1(ar202) III* |
| XB658 | *puf-8(q725)/mc6g II; der-2(tm6098) glp-1(ar202) III* |
| XB724 | *cup-2(tm2838)/hT2 I; puf-8(q725)/mc6g II; der-2(tm6098) glp-1(ar202)/hT2 III* |
| JK3182 | *gld-3(q730) nos-3(q650)/mIn1[mIs4 dpy-10(e128)] II* |
| XB686 | *cup-2(tm2838) I; gld-3(q730) nos-3(q650)/mIn1 [mIs14 dpy-10(e128)] II* |
| XB487 | *gld-2(q497) gld-1(q485)/hT2g I* |
| XB648 | *gld-2(q497) gld-1(q485) cup-2(tm2838)/hT2g I* |
| XB706 | *gld-2(q497) gld-1(q485)/hT2g I; der-2(tm6098)/hT2g III* |
| XB705 | *gld-2(q497) gld-1(q485) cup-2(tm2838)/hT2g I; der-2(tm6098)/hT2g III* |
| WU1770 | *sygl-1(am307) I* |
| XB710 | *sygl-1(am307)/hT2g I; glp-1(ar202)/hT2g III* |
| XB711 | *cup-2(tm2838) sygl-1(am307)/hT2g I* |
| XB709 | *cup-2(tm2838) sygl-1(am307)/hT2g I; glp-1(ar202)/hT2g III* |
| XB681 | *cup-2(ug1) I* |
| XB687 | *cup-2(ug1) I; ojIs23[pie-1p::GFP::C34B2.10]* |
| XB738 | *puf-8(q725)/mc6g II; glp-1(oz264) III; snx-1(tm847) X* |
| XB737 | *rrf-1(pk1417) I; puf-8(q725)/mc6g II; glp-1(oz264) III* |
