# Supplementary material for: Reduction of Derlin activity suppresses Notch-dependent tumours in the C. elegans germ line
Source: PLoS Genet. 2021 Sep 23;17(9):e1009687. doi: 10.1371/journal.pgen.1009687 (PMC8491880; doi:10.1371/journal.pgen.1009687)
Supplement: Attachment — Submitted filename: Singh et al review.pdf [file pgen.1009687.s013.pdf]

Review of Singh *et al.*, Reduction of Derlin activity suppresses Notch-dependent tumours in the *C. elegans* germ line.

This manuscript reports genetic and phenotypic data demonstrating a role for Derlin family proteins in regulating proliferation of germline stem cells in *C. elegans.* Derlin proteins function in endoplasmic-reticulum-associated protein degradation. This manuscript reports that loss of activity of the Derlin family proteins, CUP-2 and DER-2, correlates with reduced germline stem cell proliferation in sensitized *glp-1(gf)* and *glp-1(gf)* backgrounds, suggesting that these protein promote Notch signaling. This tumor suppression is specific for *glp-1(gf)* mutations, suggesting a specific effect on GLP-1/Notch signaling. Consistent with this observation, the authors show reduced expression of a transcriptional target of GLP-1/Notch, *sygl-1*, in *cup-2* and *der-2* mutants. In their various assays, the authors show that *cup-2* and *der-2* are partially redundant for function. In a *glp-1(+)* background, *cup-2* single and *cup-2;der-2* double mutants have a reduced germline stem cell population. The authors investigate some aspects of Derlin function, showing that impaired retrograde transport partially suppresses *glp-1(gf)*. Loss of Derlin function leads to accumulation of misfolded proteins and ultimately triggers the unfolded protein response. The authors tested if the UPR might be responsible for *glp-1(gf)* suppression by chemically induced ER stress and the UPR. They report that induction of ER stress in different *glp-1(gf)* backgrounds suppresses the germline tumor in a dose-dependent manner. Moreover, genetic inactivation of the ER stress response can prevent *glp-1(gf)* suppression by *cup-2*. The authors discuss several alternative scenarios for how loss of Derlin protein function might suppress the GLP-1/Notch *gf* phenotype.

The data are solid, and the conclusions are supported by multiple assays. The paper will likely be of general interest to developmental and cell biologists.

<u>Points to address</u>

Fig 3C: Distal HIM-3 signal looks more extensive in *gld-2 gld-1 cup-2;der-2* germline than in *gld-2 gld-1* control. HIM-3 may look different simply because the immunolabeling intensity is much bright in the righthand image than in the lefthand image. However, in addition, the *gld-2 gld-1 cup-2;der-2* distal germline is much narrower than the *gld-2 gld-1* distal gonad. Are these two germlines typical? The authors should show the data for distal HIM-3 labeling and comment on the distal morphology.

Page 14: The size of the *cup-2* and *der-2* proliferative zones is discussed both at the top of the page and again at the beginning of the next section. I can understand why the authors did this, but it would be helpful to the reader if to include a phrase in line 13, such as "**As noted above,** we found that *cup-2* mutant worms have a slightly smaller proliferative zone than wild-type, …"

Wording of the final paragraph of the Results is very confusing. The text talks about a proximal shift in the peak of SYGL-1 expression with increasing DTT, but the peak appears to shift <u>distally</u>. A distal shift is consistent with a smaller distal progenitor zone, and matches what's shown in Fig. 4 where the peak shifts distally in *cup-2;glp-1(gf)* relative to *glp-1(gf)*. On page 17, the authors describe this SYGL-1 expression as "shifted ~two cell diameters distally as compared to wild-type…". Yet, on page 24 they write "This proximal shift of the SYGL-1

expression curve is reminiscent of the effect of removing *cup-2* activity in *glp-1(ar202)* gonads." The authors need to go through the entire final paragraph and clarify/correct their wording.

Page 25: The upper paragraph contains a run-on sentence that should be split into 2-3 sentences.

Page 30: The authors state in the Discussion that *cup-2* mutations did not suppress *glp-1(bn18)*, yet they present data in Fig. 2 showing that the proliferative zone in *glp-1(bn18)* mutants is significantly reduced by *cup-2(tm2838)*. The authors should qualify their statement, e.g., "…as we were unable to see **strong** enhancement of the *glp-1(bn18)* loss-of-function Glp phenotype…"
