# Supplementary material for: Reduction of Derlin activity suppresses Notch-dependent tumours in the C. elegans germ line
Source: PLoS Genet. 2021 Sep 23;17(9):e1009687. doi: 10.1371/journal.pgen.1009687 (PMC8491880; doi:10.1371/journal.pgen.1009687)
Supplement: Attachment — Submitted filename: Cover Letter_Response Plos Genetics Revision_2Sep.pdf [file pgen.1009687.s014.pdf]

UNIVERSITY OF CALGARY

FACULTY OF SCIENCE

Dr. David Hansen
Department of Biological Sciences
2500 University Drive NW
Calgary, Alberta Canada T2N 1N4

T +1.403.220.7496
F +1.403.289.9311
E dhansen@ucalgary.ca

31 August, 2021

Dear PLOS Genetics Editorial Board,

Attached, please find the revised manuscript entitled "Reduction of Derlin activity suppresses Notch-dependent tumours in the *C. elegans* germ line", submitted for your consideration for publication in PLOS Genetics. We are very grateful for the comments from three expert reviewers. We have addressed all of their comments, which has significantly improved the quality of the manuscript. Attached, please find detailed descriptions of how we have addressed their comments.

Please feel free to contact me if you have any questions or concerns regarding this re-submission.

Sincerely,

Dave Hansen

UNIVERSITY OF CALGARY

Response to reviewers

Reviewer #1: Comments:

Specific Points
1. Page 5, first paragraph. Consider citing a review about genetic redundancy and robustness of developmental signaling pathways.
Thank you for this suggestion. We have included two reviews on the topic of genetic robustness in development.

2. Page 6, first paragraph. Consider citing a more recent review of ERAD. There is quite a nice one in the June 2021 issue of Molecular Cell.
Thank you for this suggestion. The review has been included in the first line on page 6.

3. Page 7, second paragraph. Consider adding an introductory figure to the main text or supplement. This will help general readers understand the experiments to simultaneously disrupt gld-1 and gld-2 function.
We have added a supplementary figure (Supp Fig 1) to serve as an introductory figure for the expected phenotype of *glp-1* and *gld-2 gld-1* disruption.

4. Page 8, last paragraph. This section may be difficult for general readers to follow without some additional explanation. Alternatively, PUF-8, it's molecular identity, and the prior findings (including those to be published elsewhere), could explained at the start of the Results section. My sense is that this portion of the Introduction, in its present form, not only disrupts the flow of the explication of the study's chief findings, but also will lose general readers. Perhaps the Introduction should focus on what was found and the surrounding context and not exactly how it was found, which can be explained in the Results section.
We have added additional information on PUF-8 for general readers, as suggested.

5. Page 10. Results section. The authors utilize a variety of alleles in their genetic analyses. Many of these are well known to the cognoscenti in the field. However, general readers and newcomers to the field might find the presentation difficult to understand. Consider putting a table in the supplement summarizing the nature of key alleles used in the genetic analyses and appropriate references.
We have included a table for the key *glp-1* alleles in Supp Table 1 and mentioned this in the beginning of the results section.

6. Page 10, last paragraph and Figure 1B. Please apply statistical analyses to the data shown in Figure 1B.
Goodness-of-fit tests such as chi-square and G-test cannot be used to compare data where the expected frequency is zero such as for comparing *puf-8; ar202* (only one category of expected frequency) to any other *ar202* genotype (two or more categories of observed frequencies) in the data. Chi-square tests have been applied to other pair-wise comparisons and have been included in the figure.

UNIVERSITY OF CALGARY

7. Page 10, first paragraph and Figures 2B and 2C. Consider reorganizing Figure 2B so the results you discuss first come first. Also cite Figures 2B and 2C as appropriate.
We have remade Fig 2B to be inline with the order of the results presented and included the *glp-1(ar202)* and *cup-2; glp-1(ar202)* results in Fig 2B.

8. Page 17, and Figure 4A. Ideally, Figure 4A should contain the wild type and cup-2 single mutants. On my computer screen, the anti-FLAG staining in Figure 4A actually looks extended in the tm2838; ar202 double.
We have added two more images in Fig 4A to show anti-FLAG staining in wild type and *cup-2* single mutants and removed the corresponding DAPI image. All images shown in Fig 4A were selected based on the fact that their measured signal intensity profile was most similar to the mean for the genotype in the graph shown in Fig 4B. Therefore, the images best represent the graph shown. While the region where anti-FLAG staining extends in Fig 4A may be similar in the *ar202* and *tm2838; ar202 double* images shown, the peak, i.e. the position where the staining looks most intense can be seen to shift more distally in the *tm2838; ar202* image compared to *ar202*. These images were also not taken at the same exposure setting so should not be directly compared for where the signal appears to die out, as this is easily influenced by the exposure setting. The position of the transition zone is more likely to be determined by the distance from the peak of SYGL-1 intensity rather than the distance from the region where the signal appears to die out by eye, as that latter is a subjective measure.

9. Page 18, second paragraph. Please refer to "unpublished data" as opposed to "data not shown."
We have included a reference to the relevant published thesis in the public domain for this information. Page 167 of the thesis shows that all three strains expressed CUP-2, as verified by a western blot.

10. Page 23, second paragraph. Is cup-2 synthetically lethal with xbp-1? If not, then the suggestion is to use an xbp-1 null allele (e.g., zc12) to complement the RNAi experiment—this is an important conclusion and an additional genetic test seems warranted.
This is a great suggestion and we agree that creating an additional strain such as *cup-2(0); glp-xbp-1(zc12) glp-1(ar202)* would provide more concrete evidence that *cup-2* requires *xbp-1* activity in order to suppress *glp-1(ar202)* tumours. The interaction between *cup-2* and *xbp-1* has not yet been tested genetically. However, we expect that *cup-2* would be synthetically lethal with *xbp-1* since *xbp-1* acts downstream of *ire-1.* It is known that *cup-2* is synthetically lethal with *ire-1* (Schaheen et al. 2009). Additionally, *xbp-1* and *glp-1* are located relatively close to each other on the same chromosome; therefore, constructing the triple mutant strain would require significantly more time than we have currently been provided to revise our manuscript. We will perform this cross with the intention of including the results in a future manuscript.

11. Page 24, second paragraph. You mean distal shift, right?
Yes. Thank you for pointing out this error in the last paragraph of the results section. We have corrected the three sentences to say distal shift.

12. Page 28, first paragraph. Is it also worthwhile considering the levels or activities of the membrane-localized enzymes that process the GLP-1/Notch receptor?

UNIVERSITY OF CALGARY

Thank you for this suggestion. We have included this possibility into the text, at the end of the section discussing the extracellular portion of the GLP-1/Notch receptor.

13. Page 31, first paragraph. The extracellular portion of the Notch receptor is luminal in the ER. The reviews I've read point out mechanistic differences in ERAD responses triggered by misfolded domains in the lumen, membrane, and cytoplasm. Might be worth a mention.
Yes, this is definitely something we have been intrigued by from the time we determined that the tumour suppression seemed to affect only Notch(gf) alleles. However, upon closer examination of the literature, it emerged that these three pathways were identified and best studied in yeast. In higher organisms, such as mammals, where ERAD complexes seem more complex and redundant, the distinction is less clear. We have mentioned this in the introduction on page 6, first paragraph. Therefore, this possibility seemed too speculative to include at this time.

14. I am not sure this is worth a mention in the Discussion, but it is perhaps worth thinking about. Previous studies from the Hansen, Schedl, and a few other labs have shown that various perturbations of the spliceosome can promote germline tumor formation. In the landmark paper on IRE1-dependent splicing of HAC1 mRNA, it was shown that defects in spliceosome-mediated splicing by themselves induce the UPR in budding yeast (Sidrauski et al., 1996). One would guess this happens in the worm, which would be a condition that would, according to the current manuscript, suppress not enhance tumor formation. No doubt that the perturbation of splicing affects multiple steps in the pathway (e.g., the gld-1 pathway) may provide some explanation. Yet, these considerations might mean that targeting ERAD or the UPR for therapeutics might be complicated and even counterproductive in some situations.

We thank the reviewer for bringing up the fact that perturbations in splicing can itself induce the UPR. We agree that this may complicate targeting the ERAD or the UPR for therapeutics. In the field of cancer therapeutics many have suggested that targeting the UPR is a double-edged sword that on one hand may make cancers more aggressive while on the other may help control it, depending on the context (Kim et al, 2015, http://dx.doi.org/10.1016/j.semcancer.2015.02.003). We speculate that part of the reason for this complexity could be due to differences in how the UPR is activated in response to different stressors. Of the three branches of the UPR, perhaps each is activated to varying degrees upon encountering different types of stresses, resulting in a net UPR response that is tailored to best address the stress. Therefore, the UPR response upon spliceosome perturbation may not be equivalent to the UPR response upon Derlin inactivation, accounting for different outcomes on tumour development. That the UPR isn't simply a binary switch and instead comes in different 'flavours' or behaves as a tunable 'rheostat' has recently been gaining traction in the field of therapeutics as well (eg. Lenghel et al. 2021. https://doi.org/10.3389/fendo.2020.614123).

Our study can help draw attention to the use of Derlin-mediated UPR induction in a specific context, i.e. Notch(gf)-dependent tumours, and will instigate further studies in other animal models to verify and expand upon our findings. We thank the reviewer for raising this important possible connection, and we have plans to investigate this further in the future.

Reviewer #2:
Points to address

1. *Fig 3C: Distal HIM-3 signal looks more extensive in gld-2 gld-1 cup-2;der-2 germline than in gld-2 gld-1 control. HIM-3 may look different simply because the immunolabeling intensity is*

UNIVERSITY OF CALGARY

much bright in the righthand image than in the lefthand image. However, in addition, the gld-2 gld-1 cup-2;der-2 distal germline is much narrower than the gld-2 gld-1 distal gonad. Are these two germlines typical? The authors should show the data for distal HIM-3 labeling and comment on the distal morphology.

Thank you for pointing out the difficulty in clearly seeing the distal HIM-3 staining and morphology in Fig 3C. We have adjusted the brightness and contrast in the image shown of the *gld-2 gld-1 cup-2; der-2* germline to make it easier to compare with the *gld-2 gld-1* germline in Fig 3C. We have also added a supplemental figure (Supp Fig 3) depicting the typical distal HIM-3 staining in these two genotypes to address this.

Yes, genotypes mutant for both Derlins, i.e. *cup-2; der-2* typically have gonads that are narrower and smaller compared to wild-type. This difference can also be seen in Fig 2. We have added a line on page 14 to draw attention to this difference.

2. *Page 14: The size of the cup-2 and der-2 proliferative zones is discussed both at the top of the page and again at the beginning of the next section. I can understand why the authors did this, but it would be helpful to the reader if to include a phrase in line 13, such as "As noted above, we found that cup-2 mutant worms have a slightly smaller proliferative zone than wild-type, ..."*

   We have added the words 'As noted above' to the statement, as suggested.

3. *Wording of the final paragraph of the Results is very confusing. The text talks about a proximal shift in the peak of SYGL-1 expression with increasing DTT, but the peak appears to shift distally. A distal shift is consistent with a smaller distal progenitor zone, and matches what's shown in Fig. 4 where the peak shifts distally in cup-2;glp-1(gf) relative to glp-1(gf). On page 17, the authors describe this SYGL-1 expression as "shifted ~two cell diameters distally as compared to wild-type...". Yet, on page 24 they write "This proximal shift of the SYGL-1 expression curve is reminiscent of the effect of removing cup-2 activity in glp-1(ar202) gonads." The authors need to go through the entire final paragraph and clarify/correct their wording.*

   We apologize for the confusion. Thank you for pointing out this error on page 24, the last paragraph of the results. We have now corrected the wording to 'distal' in accordance with the anatomical definitions of the distal/proximal ends of the germline in the three sentences of the paragraph.

4. *Page 25: The upper paragraph contains a run-on sentence that should be split into 2-3 sentences.*

   Thank you for pointing this out. This sentence has been split into three sentences.

5. *Page 30: The authors state in the Discussion that cup-2 mutations did not suppress glp-1(bn18), yet they present data in Fig. 2 showing that the proliferative zone in glp-1(bn18) mutants is significantly reduced by cup-2(tm2838). The authors should qualify their*

UNIVERSITY OF CALGARY

statement, e.g., "...as we were unable to see strong enhancement of the glp-1(bn18) loss-of-function Glp phenotype..."

We have added the word 'strong' to the statement, as suggested.

Reviewer #3:
To strengthen the overall conclusions of the study, especially in the context of the last paragraph of the Discussion, the authors could distinguish whether suppression of tumor acts on any level Notch signaling or only on the Notch gain-of-function alleles.
The genetic suppression of Notch-dependent tumors by cup-2 loss could be explained if Derlins buffered the germlines expressing temperature-sensitive glp-1 mutants from ER stress so that these genotypes were "addicted" to Derlins and sensitized to the loss of Derlin activity. This is consistent with cup-2(0) leading to a stronger reduction of Notch signaling mediated by glp-1(gf) mutants compared to the otherwise wt background.
If this were the case, loss of cup-2 would not suppress a glp-1-dependent tumor where glp-1 is wild type, so not in need of ER stress buffer, such as gld-2; teg-1 tumor (Wang et al., 2012) or gld-2 prp-17 tumor (Kerins et al., 2010). Additionally, such data would provide a stronger support to the proposal that drugs affecting Derlin activity would predominantly target tissues with aberrant rather than normal Notch signaling.

This is a very interesting idea and we appreciate the suggestion. We did test two tumourous backgrounds in which Notch is wild type –*gld-2 gld-1* and *gld-3; nos-3* (Fig 3) (Although we recognize that these tumours are not dependent on Notch activity). Since loss of Derlin activity was not able to suppress either of these tumours, we have proposed that drugs affecting Derlin activity would predominantly target Notch(gain-of-function) tumours. Additionally, the expression of the SYGL-1 readout for Notch signalling in *cup-2* mutants or upon inducing ER stress did not significantly change in Notch(wt) backgrounds (Fig 4 and Fig 8A).

We do not think that Derlins exclusively buffer only temperature-sensitive forms of Notch because we did not see a strong interaction with *glp-1(bn18)*, which is also a temperature-sensitive loss-of-function allele (Supp Table 2). We have now added a supplemental table on the nature of the Notch alleles tested in this study (Supp Table 1), to better assist readers in interpreting the relationship of Derlins to the different Notch mutants used in this study.

Since we would not be able to construct the suggested strains in the current time frame for revisions, we are unable to perform the suggested experiments at this time. It would be quite time consuming to create strains with either *gld-1* or *gld-2* since *cup-2* is located about 2.72 m.u. away from *gld-1* and 3.9 m.u. units away from *gld-2* on the same chromosome. We will perform these crosses in the future with the goal of providing the results in another manuscript.

The authors should also address the following points:

1. The title of Fig. 6 needs to be revised, the experiment in Fig. 6B does not support the conclusion that "cup-2 activity partially requires retrograde transport", as partial suppression of tumor by sorting nexin knockout doesn't speak to whether it happens through the same or different mechanism than cup-2-mediated suppression. Simply stating that "Retrograde transport might contribute to cup-2 activity" as done in the Results would be more accurate.
Additionally, the legends for panels A and B are switched, which should be corrected.

UNIVERSITY OF CALGARY

Thank you for pointing this out. This correction has been made in the legend of Fig 6B.

2. In Fig. 8B, the graph for 0mM DTT treatment doesn't have standard deviation.
All of the SYGL-1 expression plots in the main figures (Fig 4B and Fig 8) did not display the standard deviation for the negative control. This was done because plotting four standard deviation curves in Fig 4B made the plot overly-crowded and difficult to visualize, and we treated Fig 8 plots the same way for consistency. Instead, we included more detailed subplots in the supplemental figures (Supp Fig 4 and Supp Fig 6) to enable readers to more clearly see differences in standard deviation. Since we didn't state this in the figure legend it could have been confusing for readers. Therefore, we have added a line in the figure legends of Fig 4B and Fig 8 to explicitly state that the standard deviation for the negative control has not been shown in these figures but can be found in the supplemental figures.

3. I suggest modifying the color scheme of the graphs representing tumor phenotype analysis: in Figs. 1 and 6 green represents wild type germlines, while in Figs. 3, 7, and S3 green represents tumorous germlines. It would help if one color scheme were used consistently.
We agree that one colour scheme should be used consistently while referring to wild type and tumourous germlines. Thank you for pointing this out. Fig 1 and Fig 6 have now been corrected to show tumorous germlines in green.

4. In the last paragraph of the Results, the authors state that "increasing doses of DTT lead to a more proximal shift of the peak of SYGL-1 expression"… This is confusing because the Figure shows SYGL-1 shift distally (closer to the distal tip of the germline). Perhaps distal/proximal is this paragraph could be restated so that proximity to the tip of the germline is not in conflict with anatomical definitions of distal/proximal ends of germline.
Thank you for pointing out this error. We have corrected the wording to 'distal' with reference to the anatomical definition of the distal end of the germline in the last paragraph of the Results section.
